# Contrast-enhanced ultrasound (CEUS) is helpful for the characterization of solitary fibrous tumors of the pleura

**DOI:** 10.1055/a-2627-8336

**Published:** 2025-09-17

**Authors:** Ehsan Safai Zadeh, Christian Görg, Hajo Findeisen, Philip Eckwolf, Lea Dibon, Helmut Prosch

**Affiliations:** 1 Department of Biomedical Imaging and Image-guided Therapy 27271 Medical University of ViennaViennaAustria; 2 Interdisciplinary Center of Ultrasound Diagnostics, Gastroenterology, Endocrinology, Metabolism and Clinical Infectiology 61061 University Hospital of Giessen and Marburg Campus MarburgMarburgHEGermany; 3 Department for Internal Medicine 39539 Red Cross Hospital BremenBremenGermany

**Keywords:** solitary fibrous tumors of the pleura, CEUS, ultrasound, SFT, diagnosis

## Abstract

**Purpose:**

To describe the perfusion patterns of solitary fibrous tumors of the pleura (SFTP) using contrast-enhanced ultrasound (CEUS).

**Materials and Methods:**

Between November 2012 and 2024, six patients with histologically confirmed SFTP underwent B-mode ultrasound (B-US) and CEUS. Parameters from the arterial phase, including flow-in pattern, extent of enhancement (EE; marked or reduced/absent), and homogeneity of enhancement (HE; homogeneous or inhomogeneous) were retrospectively analyzed.

**Results:**

All 6 lesions displayed a flow-in perfusion pattern that originated from a peripheral point. Five lesions (83.3%) exhibited arterial hyperenhancement and 1 (16.7%) showed hypoenhancement. HE was inhomogeneous in 5 lesions (83.3%) and homogeneous in 1 (16.7%).

**Conclusion:**

On CEUS, all cases showed an arterial flow-in perfusion that originated from a peripheral point in an SFT, which could be a diagnostic clue for the non-invasive diagnosis of SFTPs. Given the risk of tumor seeding, direct surgical resection may be preferable to biopsy in these cases.

## Zusammenfassung

**Ziel:**

Beschreibung der Perfusionsmuster solitärer fibröser Tumoren der Pleura (SFTP) mittels kontrastverstärkter Ultraschall-Untersuchung (CEUS).

**Materialien und Methoden:**

Zwischen November 2012 und 2024 wurden 6 Patienten mit histologisch bestätigtem SFTP einer B-Modus-Ultraschall-Untersuchung (B-US) und einer CEUS unterzogen. Retrospektiv wurden Parameter der arteriellen Phase, einschließlich Flow-in-Muster, Ausmaß des Enhancements (EE; ausgeprägt oder reduziert/abwesend) und Homogenität des Enhancements (HE; homogen oder inhomogen), analysiert.

**Ergebnisse:**

Alle 6 Läsionen zeigten ein Flow-in-Perfusionsmuster, das von einem peripheren Punkt ausging. Fünf Läsionen (83,3%) wiesen ein arterielles Hyperenhancement auf, eine Läsion (16,7%) zeigte ein Hypoenhancement. Das HE war in 5 Läsionen (83,3%) inhomogen und in einer (16,7%) homogen.

**Schlussfolgerung:**

Bei der CEUS zeigte sich in allen Fällen ein arterielles Flow-in-Perfusionsmuster, das von einem peripheren Punkt ausging, was ein diagnostischer Hinweis auf die nicht invasive Diagnostik von SFTPs sein könnte. Aufgrund des Risikos eines Tumor-Seedings ist in diesen Fällen eine direkte chirurgische Resektion der Biopsie vorzuziehen.

## Introduction

Solitary fibrous tumors of the pleura (SFTPs) represent a rare entity, accounting for less than 5% of all pleural tumors 1 . The fewer than 2000 documented cases in the medical literature underscore the rarity of SFTPs 2 3 . SFTP tumors do not show a gender preference and manifest predominantly in the 6th and 7th decades of life, although they can occur at any age 4 5 . These mesenchymal tumors originate from subepithelial connective tissue cells of the pleura, with over 80% arising from the visceral pleura and less than 20% involving the parietal pleura, making the former significantly more prevalent 4 6 . Tumors show a range of growth patterns, presenting as pedunculated or sessile. Pedunculated forms are observed in 57–83% of cases, while sessile forms account for 17–43% 6 7 8 . Although most SFTPs are benign, approximately 10–25% exhibit malignant characteristics 2 9 10 11 .

Computed tomography (CT) is the gold standard for diagnostic imaging of SFTPs 12 . However, a diagnosis based solely on CT morphological characteristics is often challenging due to their similarity in appearance to other pathologies 5 . In contrast-enhanced CT imaging, SFTPs exhibit predominantly heterogeneous enhancement in approximately 90% of cases 13 . However, the characteristic features required to diagnose these tumors are absent 13 . Differential diagnoses for SFTPs include other mesenchymal tumors, such as leiomyosarcomas, synovial sarcomas, fibrosarcomas, and hemangiopericytomas, as well as secondary tumors such as metastases 12 14 15 . Therefore, reliable differentiation is currently possible only through histological analysis, utilizing immunohistochemical markers, such as CD34 and STAT6, and molecular pathological analysis to detect the NAB2-STAT6 gene fusion 16 . However, deciding between biopsy and direct surgical resection also remains a challenge in patients suspected of having these tumors. Currently, there are no established guidelines regarding SFT therapy and management. A 2020 meta-analysis showed that, due to inconsistencies between biopsy and resection specimens, biopsies have limited value in the preoperative evaluation of these tumors 3 . As a result, preoperative biopsy is not recommended for assessing the malignant potential of SFTPs 3 . Furthermore, anecdotal reports have suggested that biopsies of SFTPs may carry a risk of tumor cell dissemination, a phenomenon known as tumor seeding 17 .

Consequently, direct surgical resection is recommended as the method of choice, and the decision between histological verification by biopsy and direct surgical resection may be relevant for the therapeutic management of SFTPs 3 . Hence, non-invasive methods – to better characterize these tumors and potentially avoid biopsy – would be desirable.

Given their pleural location, these lesions are readily amenable to ultrasound assessment. The utility of ultrasound, and especially contrast-enhanced ultrasound (CEUS), in the evaluation of pleural lesions has previously been reported 18 19 . In recent years, CEUS has gained increasing prominence in the assessment of perfusion in a variety of pathologies, providing real-time imaging without ionizing radiation and allowing a detailed evaluation of the microvascular architecture 20 . However, data regarding the perfusion patterns of SFT in CEUS are currently limited.

The aim of this case series was to investigate the perfusion patterns of histologically confirmed SFTPs using B-mode lung ultrasound (LUS) and CEUS.

## Patients and Methods

For this retrospective case series, all patients with a histologically confirmed diagnosis of SFTP from 2 centers, who underwent contrast-enhanced ultrasound (CEUS), were included. Between November 2012 and 2024, six patients with thoracic lesions identified on CT underwent standardized B-mode ultrasound (B-US) and CEUS examinations. All ultrasound data were obtained during routine clinical procedures, in accordance with established institutional protocols. In 5 of 6 patients, ultrasound was performed to guide lesion biopsy; in one patient, it served as a sonographic correlate to the CT findings prior to surgical resection. Histopathological confirmation of the diagnosis was achieved for all patients, either through surgical resection, core-needle biopsy, or both.

Ultrasound examinations were performed using an ACUSON SEQUOIA 512 GI (Siemens, Erlangen, Germany), a GE LOGIQ E9, or a GE LOGIQ E10 (GE Healthcare, Chicago, Illinois, USA). SonoVue (Bracco Imaging S.p.A., Milan, Italy) served as the ultrasound contrast agent. A 3–6 MHz convex ultrasound probe was used. CEUS was performed in a contrast-specific, continuous mode with a low mechanical index, in accordance with the guidelines of the European Federation of Societies for Ultrasound in Medicine and Biology (EFSUMB) 20 . Patients were examined in a seated or supine position. The perfusion patterns of the lesions were continuously examined in several short examinations at 30-second intervals for up to 3 minutes. Images of the peak of enhancement of all lesions were saved.

The following B-mode ultrasound data and CEUS parameters were retrospectively analyzed.

### B-mode ultrasound

1- Echogenicity of the lesion (hypoechoic, isoechoic, hyperechoic, or complex);

2- Lesion diameter in cm.

### Contrast-enhanced ultrasound

1- Arterial flow-in perfusion pattern on CEUS;

2- Extent of enhancement (EE) at the time of peak enhancement, assessed as marked or reduced/absent 19 ;

3- Homogeneity of enhancement (HE) at the time of peak enhancement, evaluated as homogeneous or inhomogeneous; lesions displaying perfused areas alongside non-perfused regions were regarded as inhomogeneously enhanced 19 .

The enhancement of the chest wall served as an in vivo reference for assessing EE and HE. Both EE and HE were examined at the peak enhancement of the lesions.

## Results

### Demographic and clinical data

The study included 6 patients with histologically confirmed SFTP. The mean age was 65.7 years (range: 45–83 years), with 4 males (66.7%) and 2 females (33.3%). One patient (16.7%) had an underlying malignant disease at the time of diagnosis (choroidal melanoma), while the remaining 5 patients (83.3%) did not. Histological confirmation was achieved through surgical resection and core needle biopsy in 3 patients, through only core needle biopsy in 2 patients, and by only surgical resection in 1 patient.

In 2 patients, no surgical resection was performed. In 1 patient, the local tumor board recommended radiation therapy due to the patient’s poor general health. In this case, tumor progression led to compression of the left main bronchus and subsequent obstructive pneumonia, resulting in death. In this patient, the initial biopsy showed no significant nuclear atypia or mitotic activity. However, 2 years later, multiple ipsilateral and contralateral pulmonary lesions developed. A repeat biopsy revealed an SFT with 1 to 2 mitoses per 2mm ^2^ , raising suspicion of malignant behavior.

The other patient did not undergo surgical resection, as the tumor did not show any growth or progression in size, and the patient remained asymptomatic during a follow-up period of 11 years, without any evidence of tumor seeding. In the other 3 patients who underwent ultrasound-guided biopsy, no evidence of recurrence or tumor seeding was found after a follow-up period of over 3 years in all patients.

### Surgical data

In patients who underwent surgical resection, 3 tumors originated from the parietal pleura, and 1 arose from the visceral pleura. Intraoperatively, the tumors demonstrated distinct patterns of attachment and vascularization. One was attached to the mediastinum, another to the right diaphragm and thoracic wall, a 3rd to the thoracic wall at the left upper thoracic aperture, and 1 to the left lower lobe of the lung. In addition, 2 tumors were pedunculated, while the remaining 2 had a broad-based (sessile) attachment.

### B-mode LUS data

**Fig. 1:**
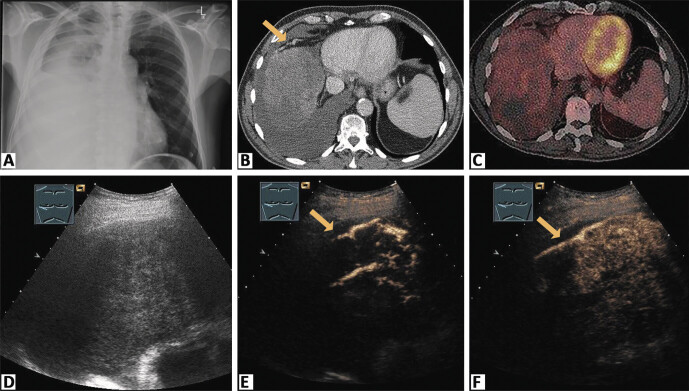
An 80-year-old male patient with a pleural-based mass in the right hemithorax. **A** Chest radiography revealed a pronounced opacity in the right hemithorax. **B** Computed tomography scans demonstrated a lesion of uncertain origin adjacent to the right lower lobe, with a feeding vessel (arrow). **C** On positron emission tomography with computed tomography, the lesion exhibited minimal fluorodeoxyglucose uptake. **D** B-mode ultrasound showed a hypoechoic, homogeneous lesion in the right hemithorax. **E** Contrast-enhanced ultrasound demonstrated a flow-in pattern originating from a peripheral point (arrow) at 16 seconds and **F** marked, inhomogeneous enhancement at 46 seconds; the feeding vessel remained visible (arrow). Histopathological examination of the resected specimen confirmed a solitary fibrous tumor of the pleura.

**Fig. 2:**
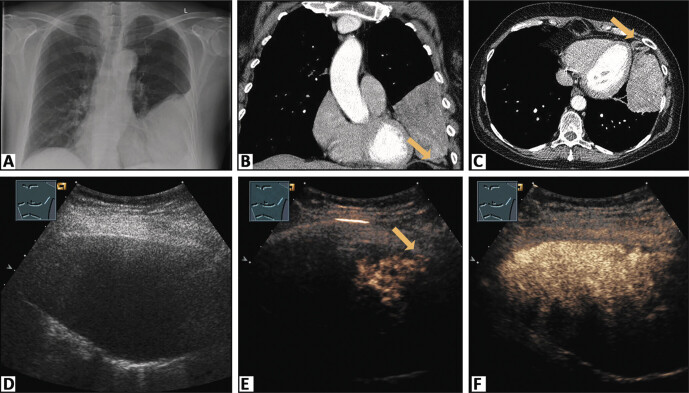
A 63-year-old female patient with a pleural-based mass in the right hemithorax. **A** Chest radiography revealed a pronounced opacity in the lower right hemithorax. **B** , **C** Computed tomography scans demonstrated a lesion of uncertain origin adjacent to the right lower lobe, with a feeding vessel (arrow). **D** B-mode ultrasound showed a homogeneous hypoechoic lesion in the right hemithorax. **E** Contrast-enhanced ultrasound highlighted a flow pattern originating from a peripheral point (arrow) at 9 seconds and **F** marked inhomogeneous enhancement at 36 seconds. Histopathological examination of the resected specimen confirmed a solitary fibrous tumor of the pleura.

**Fig. 3:**
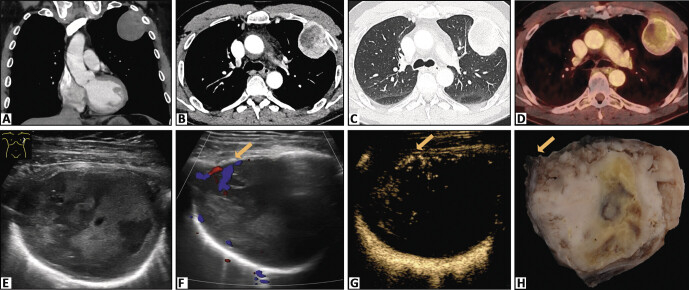
A 53-year-old male patient with a pleural-based mass in the left upper hemithorax. **A** – **C** Computed tomography scans demonstrated a pleural-based lesion of uncertain origin. **D** On positron emission tomography with computed tomography, the lesion showed moderate fluorodeoxyglucose uptake. **E** B-mode ultrasound revealed a complex echo pattern and subtle mobility of the lesion beneath the pleura, consistent with a pleura-based tumor. **F** On color Doppler sonography, a peripheral vessel could be suspected (arrow). **G** Contrast-enhanced ultrasound showed a flow pattern that originated from a peripheral point (arrow) and marked inhomogeneous enhancement at 40 seconds. **H** Histopathological examination of the resected specimen confirmed a solitary fibrous tumor of the parietal pleura with a peripheral feeding vessel (arrow).

On B-mode ultrasound, 4 lesions (66.7%) showed hypoechoic echogenicity ( Fig. 1 , Fig. 2 ), and 2 lesions (33.3%) revealed complex echogenicity ( Fig. 3 ). The mean largest lesion dimension was 9.0 cm (range: 4.5–16 cm).

### CEUS data

All lesions demonstrated a flow-in pattern that originated from a focal peripheral point ( Fig. 1 , Fig. 2 , Fig. 3 ). Five lesions (83.3%) displayed arterial hyperenhancement ( Fig. 1 , Fig. 2 , Fig. 3 ), while 1 lesion (16.7%) exhibited arterial hypoenhancement. In terms of homogeneity, 5 lesions (83.3%) showed an inhomogeneous enhancement ( Fig. 1 , Fig. 2 , Fig. 3 ), and 1 lesion (16.7%) demonstrated a homogeneous enhancement. Table 1 presents the B-US and CEUS characteristics of the study patients.

**Table:** **Table 1** Summary of B-mode ultrasound and CEUS data.

Patient number	Age	Histology	Echogenicity (B-US)	Flow-in pattern (CEUS)	EE (CEUS)	HE (CEUS)
**1**	58	Resection	Complex	From a focal peripheral point	Hyperenhancement	Inhomogeneous
**2**	83	Biopsy	Hypoechoic	From a focal peripheral point	Hyperenhancement	Inhomogeneous
**3**	63	Biopsy and resection	Hypoechoic	From a focal peripheral point	Hyperenhancement	Inhomogeneous
**4**	65	Biopsy	Hypoechoic	From a focal peripheral point	Hypoenhancement	Homogeneous
**5**	45	Biopsy and resection	Complex	From a focal peripheral point	Hyperenhancement	Inhomogeneous
**6**	80	Biopsy and resection	Hypoechoic	From a focal peripheral point	Hyperenhancement	Inhomogeneous
B-US: B-mode ultrasound; CEUS: contrast-enhanced ultrasound; EE: extent of enhancement; HE: homogeneity of enhancement; Hom: homogeneous.						

## Discussion

Solitary fibrous tumors of the pleura present a diagnostic challenge – due to their rarity and variable imaging characteristics. In this case series, we explored the role of CEUS as an additional method for non-invasive diagnosis and characterization.

A noteworthy finding was the visualization of a flow-in pattern that originated from a peripheral point in all examined cases. This observation aligns with earlier studies indicating that the presence of a major feeding vessel 14 21 on imaging, particularly on CT, is suggestive of SFTP. Unlike CT, which provides only a single snapshot of the vascular supply, CEUS permits continuous, dynamic observation of perfusion throughout the examination. This capacity to visualize and track arterial inflow over time may serve as a crucial diagnostic marker, enhancing the accuracy of non-invasive tumor identification. However, although this characteristic is considered a key feature of pleural SFT, its frequency among other pleural tumors remains unclear. Currently, no data are available regarding this characteristic in other pleural tumors.

Apart from the consistent flow-in pattern observed in all cases, the CEUS findings revealed heterogeneous vascular patterns within the tumors. Most lesions (83.3%) exhibited arterial hyperenhancement, whereas 1 case showed hypoenhancement, suggesting that perfusion patterns may vary, potentially due to differences in tumor size, histological subtype, or fibrotic content. Furthermore, the observation of inhomogeneous enhancement and non-perfused areas in 83.3% of the lesions may correspond to areas of internal necrosis, similar to patterns described in other solid tumors assessed by CEUS. The presence of necrotic and hemorrhagic areas in SFTP has been previously reported 22 .

From a clinical perspective, the ability of CEUS to clearly visualize a fill-in pattern from a focal peripheral point and to delineate the internal vascular architecture of an SFTP could have several important implications. First, if confirmed by a larger population, the presence of a fill-in pattern from a focal peripheral point may be considered a hallmark feature of SFTPs, thereby allowing clinicians to avoid biopsy and proceed directly with surgical resection in such cases, especially when no underlying malignant disease is present and the pleural tumor remains indeterminate 3 . This may enhance the preoperative differentiation of SFTPs from other pleural lesions, which range from lung carcinoma to various intrapleural sarcomas and pleural mesothelioma 12 . Second, the detailed vascular mapping provided by CEUS may guide surgical planning, enabling a more targeted approach to resection, thus potentially reducing operative complications 21 . One of the most significant complications during surgical resection is substantial hemorrhage due to the hypervascular nature of these masses. Therefore, the preoperative evaluation of the tumor’s feeding vessels is crucial for reducing complications. Some authors also recommend preoperative embolization of arterial vessels 23 24 . In this context, the use of CEUS, owing to its strictly intravascular and dynamic properties, can be helpful in addition to CT.

This case series has several limitations. It is important to acknowledge that ultrasound visualization of the pleural surface is inherently limited to approximately 70% of its area 25 . Furthermore, this study investigated only a small sample without a control group. It remains unclear how frequently a peripheral perfusion pattern that originates from a focal point occurs in other tumors. To address these limitations and given the rarity of SFTPs, further prospective multicenter studies are essential.

## Conclusion

In conclusion, this case series demonstrates the potential value of CEUS in the diagnostic workup of SFTPs. The ability to visualize a peripheral perfusion pattern that originates from a focal point suggests that CEUS may serve as a helpful, non-invasive diagnostic tool. If a peripheral punctate perfusion pattern can be reliably detected by CEUS in pleural lesions, particularly in patients without underlying malignant or systemic disease, the possibility of an SFTP should be considered. Given the risk of tumor seeding, direct surgical resection may be preferable to biopsy in these cases. However, it remains unclear how specific this finding is to SFTPs and how frequently it may occur in other pleural tumors. A prospective multicenter study involving a large patient cohort with various pleural tumors is, therefore, warranted.
